# Practical cut-off value for radon concentration in indoor air using an activated-charcoal radon collector

**DOI:** 10.1093/rpd/ncae108

**Published:** 2024-11-14

**Authors:** Yumi Yasuoka, June Takemoto, Yasutaka Omori, Nanaho Kawamoto, Nao Goda, Hiroyuki Nagahama, Jun Muto, Shinji Tokonami, Masahiro Hosoda, Takeshi Iimoto, Takahiro Mukai

**Affiliations:** Kobe Pharmaceutical University, 4-19-1 Motoyamakitamachi, Higashinada-ku, Kobe City, Hyogo 658-8558, Japan; Kobe Pharmaceutical University, 4-19-1 Motoyamakitamachi, Higashinada-ku, Kobe City, Hyogo 658-8558, Japan; Hirosaki University, 66-1 Hon-cho, Hirosaki City, Aomori 036–8564, Japan; Kobe Pharmaceutical University, 4-19-1 Motoyamakitamachi, Higashinada-ku, Kobe City, Hyogo 658-8558, Japan; Kobe Pharmaceutical University, 4-19-1 Motoyamakitamachi, Higashinada-ku, Kobe City, Hyogo 658-8558, Japan; Department of Earth Science, Graduate School of Science, Tohoku University, 6-3, Aoba, Aramaki, Aoba-ku Sendai, Miyagi 980-8758, Japan; Department of Earth Science, Graduate School of Science, Tohoku University, 6-3, Aoba, Aramaki, Aoba-ku Sendai, Miyagi 980-8758, Japan; Hirosaki University, 66-1 Hon-cho, Hirosaki City, Aomori 036–8564, Japan; Hirosaki University, 66-1 Hon-cho, Hirosaki City, Aomori 036–8564, Japan; Division for Environment, Health, and Safety, The University of Tokyo, 7-3-1 Hongo, Bunkyo-ku, Tokyo 113-8654, Japan; Kobe Pharmaceutical University, 4-19-1 Motoyamakitamachi, Higashinada-ku, Kobe City, Hyogo 658-8558, Japan

## Abstract

The World Health Organization (WHO) recommends that countries adopt the reference level of 100 Bq m^−3^ for indoor radon gas. Adopting the reference level requires a preliminary assessment of the indoor radon concentration. In the preliminary investigation, a cut-off value is useful, which is a value for selecting samples that can be reliably determined to be below the reference level (in this paper, the WHO reference level) using a straightforward method. If the true value was the WHO reference level, then the cut-off value of the PicoRad collector for selecting samples was determined as 80 Bq m^−3^ through the analysis of the 95% prediction interval.

## Introduction

Radon (^222^Rn) is transported from the soil into the air and is present outdoors and indoors [1]. High levels of indoor radon exposure are a significant cause of lung cancer. Darby et al. [2] reported that exposure to radon increases the risk of lung cancer by 16% per 100 Bq m^−3^. The World Health Organization (WHO) recommends that countries adopt the reference level [3] of 100 Bq m^−3^ for indoor radon gas.

Adopting the reference level requires a preliminary assessment of the indoor radon concentration. The preliminary survey aimed to identify the dwellings that may have exceeded the reference level. The United States Environmental Protection Agency (US EPA) has established radon standards of practice [4]. One of the most common methods for preliminary radon surveys is using an activated carbon collector [5, 6], which the US EPA introduced as a passive-type device for short-term testing. A PicoRad collector (PicoRad: AccuStar Labs, USA) [7] was used as an activated carbon collector [6]. The PicoRad is a plastic vial containing activated carbon and a desiccant. It is an inexpensive measurement device that can collect radon data from multiple locations. Radon was adsorbed onto the micropores of the activated carbon depending on the concentration difference between the tested air and micropore air. The PicoRad is sensitive to variations in radon concentration and is available for short-term measurements. Therefore, it measured exposure values for 2 days under closed-room conditions [7].

Zhukovsky et al. [8] reviewed 63 national and regional indoor radon surveys in kindergartens and schools using the PicoRad. The PicoRad is expected to be applied to the regular check of exhaust monitors at radioisotope facilities using indoor radon [9], and it is also expected to be used to measure radon exhalation rates from the ground and high-level atmospheric radon concentrations [1, 10, 11]. Recently, methods using 95% (or 99%) prediction intervals (*PI*) have been proposed to determine cut-off values for screening samples that are unlikely to exceed a standard value for contaminants in food [12–15]. Like food testing, determining measurement values that are expected to be reliably below a reference level helps implement measures to reduce exposure to indoor radon. The cut-off value was used to select samples reliably determined to be below the reference value (the WHO reference level in this study) using a simple method [12–15].

In this study, the valid radon concentrations of the PicoRad were determined based on the radon standard of practice of the US EPA [4]. Next, we calculated the range of the PicoRad results using the 95%*PI* when the true value was the WHO reference level. We identified the cut-off value, which was the PicoRad value guaranteed to be lower than the WHO reference level.

## Methods

**Figure 1:**
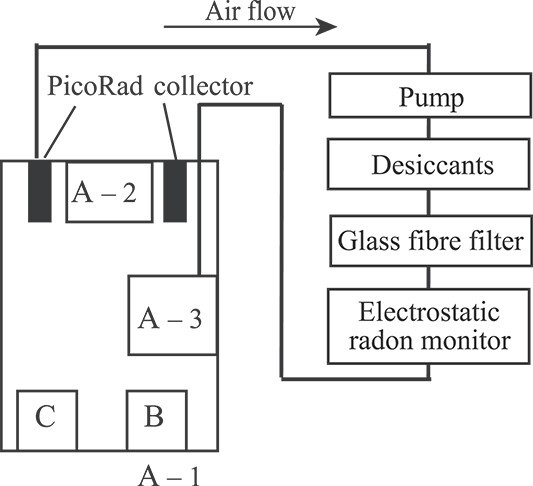
Scheme of the measurement system using the PicoRads [16]. (A) Thermo recorder (A–1: measurement in the room, A–2: measurement in the chamber, A–3: measurement of air from the electrostatic radon monitor in the system), (B) radon source, (C) humidifier.

We exposed the four PicoRads placed in a homemade radon accumulation chamber (12-L polypropylene box) for 2 days (Fig. 1) [16]. Groundwater (radon concentration: approximately 230 kBq m^−3^) [17] was diluted with tap water and used as the radon source. Temperature, relative humidity, and barometric pressure data were recorded using a 3-channel data logger (Thermo Recorder TR-73U, T&D Corporation, Japan). Air was circulated using a pump at a rate of 1 L min^−1^. Air was dried using a desiccant (CaSO_4_, W.A. Hammond Drierite Co., Ltd., USA). After removing the radon progeny nuclides in the air with a glass fibre filter (Bertin Technologies, France), the radon concentration was measured using an electrostatic collection radon monitor (PMT-TEL, Pylon Electronics Inc., Canada, detector: ZnS(Ag) scintillation counter). The relative humidity inside the radon accumulation chamber was controlled at 32–74% [16, 18].

The true radon concentration was the mean value with standard deviation *C*_S_ ± *σ*_S_ (Bq m^−3^), measured with the electrostatic collection radon monitor. The mean value of the counting rate over the last 6 h of exposure (6 h before collecting the PicoRads) was converted into the radon concentration [16, 19]. The calibration factor was determined at the radon exposure system in the Institute of Radiation Emergency Medicine, Hirosaki University [20] (see the Appendix). The background value was determined by passing nitrogen gas through the electrostatic collection radon monitor. For example, when the diluted groundwater with a radon concentration of 45 kBq m^−3^ was set as the radon source in Fig. 1, *C*_S_ was 77 Bq m^−3^.

Furthermore, after exposure, 15 mL of liquid scintillator (Insta-Fluor Plus, PerkinElmer Inc., USA) was poured into the PicoRads. The radon adsorbed on the activated charcoal in the PicoRad was sufficiently eluted into a liquid scintillator. PicoRad was analysed for 60 min using a liquid scintillation counter (Tri-Carb 2300TR; Packard Instrument Co., Inc., USA). The mean count rate of the four PicoRads measured using a liquid scintillation counter was converted into radon concentration using a previously reported formula [16, 19, 21] (see the Appendix). This radon concentration with standard deviation obtained by the PicoRads was used as the PicoRad value *C*_P_ ± σ_P_ (Bq m^−3^). We confirmed that there was no bias in the values of the four PicoRads installed in the homemade radon accumulation chamber and assumed that the *C*_P_ values were normally distributed.

## Results

In total, 27 experiments were conducted in the radon concentration range of 38–114 Bq m^−3^. For all of the data, the relative standard deviation of the mean of the four PicoRads was lower than 15%, thus meeting the US EPA requirement [4, 5]. The relative percent difference [5] *RPD*% ± *σ*_R_ and its 95%*CI*_R_ (95% confidential interval) were calculated using Equations (1) and (2), respectively:


(1) \begin{align*} RPD\% & \pm{\sigma}_{\mathrm{R}}=\frac{100\left({C}_{\mathrm{P}}-{C}_{\mathrm{S}}\right)}{C_{\mathrm{S}}} \nonumber \\{}& \pm \frac{100\left({C}_{\mathrm{P}}-{C}_{\mathrm{S}}\right)}{C_{\mathrm{S}}}\sqrt{\frac{{\sigma_{\mathrm{P}}}^2+{\sigma_{\mathrm{S}}}^2}{{\left({C}_{\mathrm{P}}-{C}_{\mathrm{S}}\right)}^2}+{\left(\frac{\sigma_{\mathrm{S}}}{C_{\mathrm{S}}}\right)}^2}, \end{align*}

(2) \begin{equation*} 95\%C{I}_{\mathrm{R}}= RPD\%\pm{k}_{\mathrm{R}}{\sigma}_{\mathrm{R}}\sqrt{\frac{1}{n_{\mathrm{R}}}}, \end{equation*}

where

*n*_R_ is number of data points *n*_R_ = 4,

*k*_R_ is the *t*-value for 95% level of confidence

(two-sided test; *df* = 3); *k*_R_ = 3.18.

**Figure 2:**
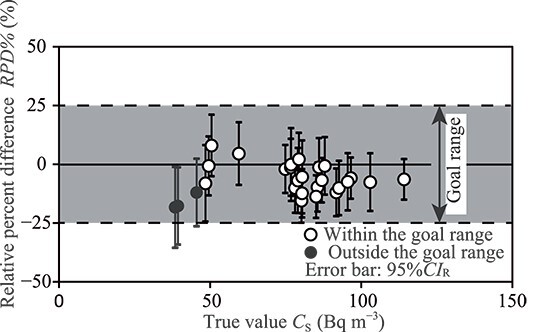
Assessment of the PicoRads based on the relative percent difference, the *RPD*% of the goal range is between −25 and 25%.

The *RPD*% of the US EPA’s goal for agreement [5, 6] (goal range) is between −25 and 25%. Figure 2 shows the relationship between *C*_S_ and *RPD*% values. When the 95%*CI*_R_ of *RPD*% (error bars in Fig. 2) was within the goal range (i.e. the grey zone in Fig. 2), the PicoRad values between 48 and 114 Bq m^−3^ met the validity of the radon data.

## Discussions

**Figure 3:**
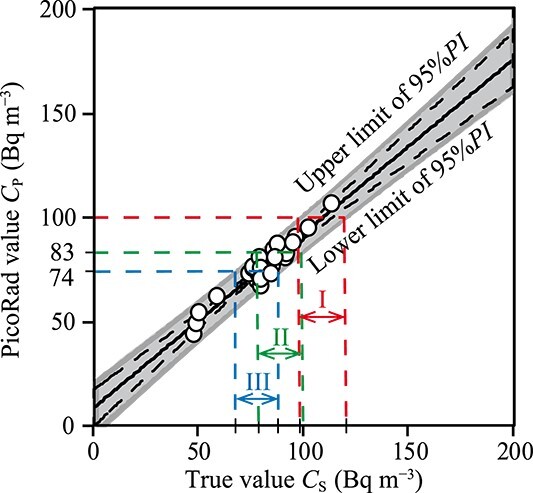
The 95%*PI* of the PicoRad for radon concentration [16]; based on these data and linear regression (black line), a 95%*CI* (dashed lines) and a 95%*PI* (grey zone) were determined by Equations (3) and (4), respectively; open circles: data within the goal range in Fig. 2.

The cut-off value of PicoRad was determined using radon data ranging from 48 and 114 Bq m^−3^, which met the validity based on the results shown in Fig. 2. A correlation scatter plot of the radon concentration, comparing *C*_S_ and *C*_P_, is shown in Fig. 3. Based on regression analysis applied to these data (Fig. 3), a 95%*CI* and a 95%*PI* of *C*_P_ were determined by Equations (3) and (4), respectively [16]:


(3) \begin{equation*} 95\% CI=A{C}_{\mathrm{S}}+B+{k}_{\mathrm{\varepsilon}}{\sigma}_{\mathrm{\varepsilon}}\sqrt{\frac{1}{n_{\mathrm{\varepsilon}}}+\frac{\left({C}_{\mathrm{S}}-\overline{C_{\mathrm{S}}}\right)}{S_{C_{\mathrm{S}}{C}_{\mathrm{S}}}}}, \end{equation*}

(4) \begin{equation*} 95\% PI=A{C}_{\mathrm{S}}+B+{k}_{\mathrm{\varepsilon}}{\sigma}_{\mathrm{\varepsilon}}\sqrt{1+\frac{1}{n_{\mathrm{\varepsilon}}}+\frac{\left({C}_{\mathrm{S}}-\overline{C_{\mathrm{S}}}\right)}{S_{C_{\mathrm{S}}{C}_{\mathrm{S}}}}}, \end{equation*}

where.

*A*: slope of regression curve *A* = 0.838,

*B*: intercept of regression curve *B* = 8.32 (Bq m^−3^),

*n*_ε_: number of data *n*_ε_ = 24,

*df*: degrees of freedom *df* = *n*_ε_ − 2 = 22,

*k*_ε_: *t*-value for 95% level of confidence,

(two side test; *df* = 22), *k*_ε_ = 2.07,

*σ*_ε_: standard error of the estimate, *σ*_ε_ = 4.02 (Bq m^−3^),

$ {S_{C_{\mathrm{S}}{C}_{\mathrm{S}}}} $ : Sum of the squared deviations,

$ {S_{C_{\mathrm{S}}{C}_{\mathrm{S}}}} = 5.96 \times 10^3 $ (Bq m^−3^)^2^,

$ \overline{C_{\mathrm{S}}} $ : mean of *C*_S_ (Bq m^−3^) $ \overline{C_{\mathrm{S}}} =80.8 $ (Bq m^−3^).

We used the 95%*PI* in Fig. 3 to determine the relationship between the true value of *C*_S_ and the cut-off value of the PicoRad value $ {C_{\mathrm{P}}} $. When the PicoRad value $ {C_{\mathrm{P}}} $ was 100 Bq m^−3^ (the WHO reference level), the true radon concentration $ {C_{\mathrm{S}}} $ was in the range I in Fig. 3 within the 95%PI of the $ {C_{\mathrm{S}}} $. The range I was 99 to 121 Bq m^−3^ may exceed 100 Bq m^−3^. When the *C*_P_ was 83 Bq m^−3^, the true radon concentration $ {C_{\mathrm{S}}} $ was in the range II in Fig. 3 within the 95%*PI* of the $ {C_{\mathrm{S}}} $. The range II was 79–100 Bq m^−3^, reducing the risk of over 100 Bq m^−3^ to 5%. Therefore, the cut-off value, which was used to select samples reliably determined to be below the reference value, was determined to be 83 Bq m^−3^. In conclusion, the cut-off value of PicoRad was determined to be 80 Bq m^−3^ to round down.

Finally, we provide a practical application to the USA.

EPA’s radon standards of practice that allow activated carbon collectors like the PicoRad to be used in preliminary radon studies. The US EPA [5, 6] proposed that the action level for indoor radon concentration is 148 Bq m^−3^ or greater. Moreover, the US EPA suggested repairing the building if test results indicate radon concentrations are between 74 and 148 Bq m^−3^. This lower range limit (74 Bq m^−3^) corresponds to the cut-off value in this paper. When the PicoRad value *C*_P_ is 74 Bq m^−3^, the true radon concentration $ {C_{\mathrm{S}}} $ was in the range III in Fig. 3 within the 95%*PI* of the *C*_S_. The range III was 68–89 Bq m^−3^, and we found that it did not exceed 100 Bq m^−3^ (the WHO reference level).

## Supplementary Material

APPENDIX_ncae108
