# Supplementary material for: Practical cut-off value for radon concentration in indoor air using an activated-charcoal radon collector
Source: Radiat Prot Dosimetry. 2024 Nov 14;200(16-18):1701–5. doi: 10.1093/rpd/ncae108 (PMC11561575; doi:10.1093/rpd/ncae108)
Supplement: APPENDIX_ncae108 [file appendix_ncae108.docx]

**APPENDIX**

**Equipment calibration**

Using the radon exposure system (radon concentration:1800 Bq m^−3^) at the Institute of Radiation Emergency Medicine, Hirosaki University^(20)^, a calibration factor of 1.07 was determined for an AlphaGUARD (Genitron GmbH, Germany). Using this AlphaGUARD, the electrostatic collection radon monitor's conversion coefficient (0.483 cpm m^3^ Bq^−1^) was determined at a radon concentration of 1000 Bq m^−3^. The conversion coefficient (around 13 cps m^3^ kBq^−1^) was determined at a radon concentration of around 300 Bq m^−3^ for the PicoRads using this electrostatic collection radon monitor. The PicoRads were measured using a liquid scintillation counter to determine the net count rate. After performing radon decay correction, temperature correction, radon adsorption correction, and correction of the radon elution rate to the scintillator for this net count rate, the radon concentration was calculated using the conversion coefficient.
